# A Comparative Study of the Use of Stratified Cross-Validation and Distribution-Balanced Stratified Cross-Validation in Imbalanced Learning

**DOI:** 10.3390/s23042333

**Published:** 2023-02-20

**Authors:** Szilvia Szeghalmy, Attila Fazekas

**Affiliations:** Faculty of Informatics, University of Debrecen, H-4028 Debrecen, Hungary

**Keywords:** imbalanced learning, cross validation, SCV, DOB-SCV

## Abstract

Nowadays, the solution to many practical problems relies on machine learning tools. However, compiling the appropriate training data set for real-world classification problems is challenging because collecting the right amount of data for each class is often difficult or even impossible. In such cases, we can easily face the problem of imbalanced learning. There are many methods in the literature for solving the imbalanced learning problem, so it has become a serious question how to compare the performance of the imbalanced learning methods. Inadequate validation techniques can provide misleading results (e.g., due to data shift), which leads to the development of methods designed for imbalanced data sets, such as stratified cross-validation (SCV) and distribution optimally balanced SCV (DOB-SCV). Previous studies have shown that higher classification performance scores (*AUC*) can be achieved on imbalanced data sets using DOB-SCV instead of SCV. We investigated the effect of the oversamplers on this difference. The study was conducted on 420 data sets, involving several sampling methods and the DTree, kNN, SVM, and MLP classifiers. We point out that DOB-SCV often provides a little higher *F*1 and *AUC* values for classification combined with sampling. However, the results also prove that the selection of the sampler–classifier pair is more important for the classification performance than the choice between the DOB-SCV and the SCV techniques.

## 1. Introduction

One of the crucial challenges in machine learning and data mining is how to achieve the desired classification accuracy when handling data with significantly skewed class distributions. In such a case, the accuracy of the classification of the majority class elements (samples) is better than the classification of the minority class samples in most cases. This challenge eventually led to “learning from imbalanced data”, the birth of a new field of statistical learning.

The importance of this research area is continuing to grow because the problem can appear almost anywhere where the data belongs to more than one category. A popular example from the financial field is bank card fraud detection [1,2], where there is a strong imbalance to deal with since the number of frauds is negligible compared to regular transactions. From the medical field, countless diagnostic tasks could be mentioned [3,4] where the data sets often do not adequately represent reality. For example, due to data collection costs or because healthy individuals cannot be included in the studies for ethical reasons. Finally, from the industrial field, it is worth mentioning fault detection [5], and from the area of human–machine interaction, we would highlight gesture, emotion [6] recognition and the various areas of activity recognition [7,8,9], sports [10], gaming [11], and fall detectors [12,13], which are primarily, but not exclusively, used in elderly monitoring systems or medical applications.

There are roughly two main approaches for handling the imbalance problem at the data level—undersampling and oversampling. Undersampling techniques are based on removing samples from the majority class, but it can lead to information loss. The oversamplers augment the data sets with replicas of the minority samples or with similar synthetic ones. Oversampling usually gives a better result than undersampling [14], but it should be kept in mind that if the degree of overlap between the two classes increases during the process, it can make the classification more difficult. For the sake of completeness, it is worth mentioning that the two above-mentioned approaches can be combined (i.e., deleting unnecessary elements from the majority set and generating new synthetic elements into the minority set), which can be called a hybrid method [15].

In a real application, it is very important to know about the performance of the given classifier. In order to validate the performance, the so-called k-fold cross-validation is one of the most commonly used methods in the literature. The basic idea of this method is to split the elements into *k* groups randomly. Nevertheless, the problem of data shift can weaken the validation [16]. If we randomly sample from the majority and minority classes according to the original distribution, we can achieve a more robust validation [17] since the distributions of the partitions (*folds*) will be similar to the initial distribution. This method is the so-called stratified cross-validation (SCV) [18]. However, the problem of covariate shift still can appear. The interested reader can find more details about SCV and its limits in Ref. [19].

A validation technique called “Distribution optimally Balanced SCV” (DOB-SCV) can be used to avoid the covariate shift problem. The main idea of this validation is to select the closest neighbors and put them in different folds to keep the distribution in the folds close to the original distribution [20].

In this paper, we investigate the SCV and the DOB-SCV methods. The difference between the two techniques is known from the literature [20], but there is a lack of extensive studies on the performance of different sampler–classifier combinations when using DOB-SCV instead of SCV for validation. To the best of our knowledge, the most exhaustive study between DOB-SCV and SCV was carried out by Lopez et al., but only one oversampler, the SMOTE, was used to balance the data sets [19].

To design a complex experiment, we selected the commonly used oversampling methods (Section 2.3), classifiers (Section 2.2), and data sets (Section 2.4) from the literature, and we also generated synthetic data sets to be able to observe the effect of clusters within classes. However, we did not involve deep learning networks because our study primarily focuses on small and medium-sized data sets, where the minority set contains few samples not only in comparison to the majority set but also in absolute terms.

The rest of this paper is organized as follows. Section 2 describes the research methodology for this study. Section 3 presents the results of our complex experiments. Finally, Section 4 presents the conclusions of this paper.

## 2. Materials and Methods

In this section, we summarize the basic concepts necessary to understand the results of the experiments. Interested readers can read about the validation methods, the classifiers and samplers involved in the study, and the data sets and measures used.

### 2.1. Validation Methods

The methods that are the subject of our study were developed from the *k-fold cross-validation*. This method randomly shuffles the samples of the data set and divides them into *k* parts (*folds*) of (nearly) equal size. Then, for each fold *f*, the other *k* − 1 folds are used to train a classifier, and the fold *f* is used to validate the obtained model according to a suitable measure (Section 2.5). The performance of the model is considered to be the mean of the validation results across the iterations. As we mentioned in the Introduction, one of the well-known disadvantages of this solution is that the proportion of samples belonging to different classes can vary significantly for the entire data set and for the folds. In addition, there is a risk—and this risk is high for severely imbalanced data sets—that some of the folds do not contain elements from all classes. Therefore, it is recommended to repeat the procedure a few times and to average the results to get a more accurate estimate of the model’s performance [21].

#### 2.1.1. SCV

The stratified k-folds cross-validation splits the data set on *k* folds such that every fold has (nearly) the same percentage of samples from minority and majority classes as the complete set. One fold is selected for the test, and the rest is used for training (Algorithm 1). The further steps of the procedure are the same as for cross-validation.
**Algorithm 1** Fold generation of SCV (based on Ref. [20])**Require:** *k*            *// number of folds***Require:** $C=\{C_{1},C_{2},\ldots ,C_{n}\}$   *// classes***Ensure:** $F_{1},F_{2},\ldots ,F_{k}$                         *// generated folds*




$F_{1}\leftarrow \emptyset ,F_{2}\leftarrow \emptyset ,\ldots ,F_{k}\leftarrow \emptyset$


**for** i:=1 to *n* **do**



      $n\leftarrow \lfloor count\left(C_{i}\right)/k\rfloor$



      **if** $i\leq (count\left(C_{i}\right)$ mod *k*) **then**



            n←n+1



      **end if**



      **for** j:=1 to *k* **do**



            S← randomly select *n* samples from $C_{i}$



            $F_{j}\leftarrow F_{j}\cup S$



            $C_{i}\leftarrow C_{i}$ \*S*



      **end for**



**end for** 


#### 2.1.2. DOB-SCV

Zen and Marinez pointed out that the distribution of the folds (and thus the training and test sets formed from them) can change strongly even if the proportions of samples from different classes are preserved by the SCV, especially if the number of samples in one or more classes is small. They suggested that nearby points belonging to the same class should be placed in different folds [22], but the goal is better achieved by the DOB-SCV proposed by Moreno-Torres et al. The DOB-SCV moves a randomly selected sample and its *k* nearest neighbors into different folds and repeats this until the samples from the original set run out (Algorithm 2). After partitioning the data set, the procedure continues as specified for cross-validation.
**Algorithm 2** Fold generation of DOB-SCV (based on Ref. [20])**Require:** *k*             *// number of folds***Require:** $C=\{C_{1},C_{2},\ldots ,C_{n}\}$     *// classes***Ensure:** $F_{1},F_{2},\ldots ,F_{k}$                           *// generated folds*





$F_{1}\leftarrow \emptyset ,F_{2}\leftarrow \emptyset ,\ldots ,F_{k}\leftarrow \emptyset$


**for** i:=1 to *n* **do**


      **while** $count(C_{i})>0$ **do**


            $x_{1}\leftarrow$ randomly select sample from $C_{i}$


            $F_{1}\leftarrow F_{1}\cup \left\{x_{1}\right\}$


            $C_{i}\leftarrow C_{i}$ \ $\left\{x_{1}\right\}$


            **for** j:=2 to *k* **do**


                 $x_{2}\leftarrow$ select the nearest neighbour of $x_{1}$ from $C_{i}$


                 $F_{j}\leftarrow F_{j}\cup \left\{x_{2}\right\}$


                 $C_{i}\leftarrow C_{i}$ \ $\left\{x_{2}\right\}$


                 **if** $count(C_{i})=0$ **then**


                      j←k                         *// end for j*


                 **end if**


            **end for**


      **end while**


**end for** 


### 2.2. Classifiers

In this section, we briefly introduce the classification procedures involved in the experiment.

#### 2.2.1. kNN

The k-nearest neighbor classifier (kNN) is a very simply supervised machine learning method. The classification of each sample is based on the vote of its nearest neighbors. All neighbors vote that the element belongs to their class. In the simplest case, the decision is made by majority rule, but it is also customary to weight the votes by the reciprocal of their distance from the sample to be classified. It is easy to see that this learning method is sensitive to the training set and selection of metrics. More detailed information can be found in Refs. [23,24].

#### 2.2.2. SVM

The support vector machine (SVM) is a supervised machine learning method developed by V. Vapnik. The main idea of this classifier is to find the margins between two classes based on the support vectors determined from the training set. In the linear case, the separating hyperplane (whose task is to separate the samples of the different classes) can be determined based on the support vectors by maximizing their distance from the margins. More detailed information can be found in Refs. [24,25].

#### 2.2.3. MLP

Multi-Layer Perceptron (MLP) is a neural network with hidden layers and backpropagation training. One of the important advantages of this classifier is its ability to classify non-convex sets. However, finding the global optimum is not guaranteed. More detailed information can be found in Ref. [26].

#### 2.2.4. DTree

The decision tree is one of the well-known classifiers that reduces complicated decisions to a series of simple ones. The trained model can be considered a tree-shaped flowchart of elementary decisions, where the vertices have an attribute, the edges between the vertices with the result of this test (with the corresponding value of the given attribute) are labeled, while the leaf tips the decision itself (that is, the value of the attribute representing the corresponding class) [24,27].

### 2.3. Oversamplers

The research was carried out using sampling methods that have proven their effectiveness on a wide range of imbalanced data sets [28], and the SMOTE, which was included in the test because it is widely used, despite its known drawbacks, and forms the basis of many more effective methods. In this section, we present the chosen methods briefly.

The SMOTE (Synthetic Minority Over-sampling Technique) [29] generates new samples along the segments connecting a minority sample (*seed*) with its nearest minority neighbors (*co-seeds*). Unfortunately, some synthetic samples may be more similar to the majority samples than to the minority ones, which may harm the classification. The SMOTE-TomekLink [30] handles this problem by a post-filtering step, which searches for pairs of samples whose members belong to different classes but are closer to each other than to any of the elements of their own class. The majority sample of such pairs is deleted. The SMOTE-IPF [31] also applies post-filtering, deleting samples from the data set whose classification is not clear based on the votes of the members of an ensemble classifier. While according to Lee’s method, the samples generated in the wrong location should be rejected immediately [32]. CCR [33] uses a less drastic solution, cleaning the environments of the minority samples by pushing the nearby majority ones outside a circle with a certain radius. The new synthetic samples are generated around the minority samples in these “clean” environments. Other methods focus on the proper selection of seeds and co-seeds used to generate synthetic samples. The Assembled-SMOTE [34] connects minority samples close to the decision boundary with minority samples further away from it, reducing the chance of the new samples falling among the majority ones. The ProWSyn attempts to generate the right amount of synthetic samples around the minority elements, considering how far they are from the decision boundary [35]. The purpose of SMOBD [36] is similar, but it decides the number of samples to generate based on the estimated density of the samples. LVQ-SMOTE [37] uses a special method because the synthetic samples are derived from reference data sets. The selection of new samples is based on the similarity of the codebooks of the reference data sets and the data set to be oversampled. For the experiments, we decided to use a version that, instead of reference data sets (there are no guidelines for choosing them), generates the codebooks based on the minor set of the data set to be oversampled [38]. The G-SMOTE combines the two principles. It detects outliers based on a Gaussian mixture model (GMM) to keep the number of samples generated near them low, but it also uses GMM to reject synthetic samples that fit more into the majority class than the minority class. The success of the polynomial fitting method lies in the different sampling strategies. By choosing the star topology, new samples are created between the original minor samples and the center of the minority class. In the case of mesh and bus topology, new samples can be created between any two minority samples and the neighboring minority samples, respectively, while the polynomial curve topology generates the synthetic samples along a “trend curve”. (In the following, we will refer to these methods as *named samplers*).

In addition, we also included automatically generated oversamplers in the study, some of which do not use clustering, some of which use DBSCAN clustering [39], and some of which categorize the samples into Border-Safe-Noise sets. The Border and Safe sets usually include samples close to the decision border and far from it if they do not appear to be noise. Different weighting strategies were used to select the seed and co-seed points. Details can be found in Ref. [40]. With the help of the resulting significant number of samplers (460), we intended to test whether the difference between the two validation methods can be observed even in the case of different sampling strategies.

### 2.4. Data Sets

We examined the effect of the two validation techniques on data set collections of the KEEL repository [41,42], which are specifically recommended for investigating imbalanced classification problems. The collections contain diverse data sets taken from real life, from the field of life science (e.g., Abalone [43], Ecoli [44], Dermatology [45]), decision-making (e.g., Car Evaluation [46]), quality inspection (e.g., Wine Quality [47]), object-recognition (e.g., Statlog [48]), etc. The category variables with two unique values were transformed by label encoding, and the category variables with more than two values by one-hot coding.

To observe the effect of within-class clusters, we also generated synthetic data sets using scikit-learn [49]. Each data set contains 600 samples with either 4 or 8 features. The *minority* and the *majority* classes are composed of 1–4 clusters. The samples were randomly drawn from a normal distribution around the vertices of a *D*-dimensional hypercube, where *D* is the number of features. The length of the sides, which affects the separability of the classes, was set to 1.4. The imbalance ratio (ratio of the number of majority and minority samples) is around 8 and 16 (for simplicity, we will refer to these as IR8 and IR16 data sets.) A total of 320 data sets were created.

According to our experience, these values result in data sets that cannot be classified too easily with the classification methods included in the study, which is important because neither the effect of the oversamplers nor the effect of the validation methods could be observed in easy problems.

### 2.5. Measures

There are several measures for evaluating the performance of classifiers [50], but many of them are sensitive to the difference between the size of the classes, so the degree of imbalance must also be considered for their proper interpretation. For example, if the imbalance ratio is high, accuracy (1) is determined almost exclusively by the success of the classification of the majority class samples [15]. On the other hand, if the performance has to be characterized by a single value, the use of *F*1-scores (3), the area under the ROC curve $AUC_{ROC}$ (it can be estimated easily by using the trapezoidal-rule), which is considered robust even against imbalance [51] or perhaps *G*-mean scores (4), is typical for imbalanced data sets.

(1) $$Acc=\frac{TN+TP}{TN+TP+FN+FP}\;,$$

(2) $$AUC=\frac{1+\frac{TP}{TP+PN}-\frac{FP}{FP+TN}}{2}$$

(3) $$F1=\frac{2TP}{2TP+FN+FP}\;,$$

(4) $$G=\sqrt{\frac{TP}{TP+FN}\cdot \frac{TN}{TN+FP}}\;,$$

The evaluation tool we used [38] defines the *TP* (true positive) and *TN* (true negative) as the total number of correctly classified minority and majority samples, respectively, over the validation folds. Similarly, the *FN* (false negative) and *FP* (false positive) are defined as the total number of wrongly classified minority and majority samples. In this paper, we primarily use *F*1 and *AUC* scores.

## 3. Experiments and Results

The experiment was conducted as follows. First, we created the training and test sets required for the 5-fold, three-repeat SCV and DOB-SCV methods for each KEEL and synthetic data set mentioned in Section 2.4, for which we used the scikit-learn package [49]. Next, we applied all the oversamplers to the training sets independently. The samplers mentioned by their name in Section 2.3 were used with all the parameter combinations specified in the smote-variants package [38]. The artificially generated samplers were run with their default parameterization.

Then, we trained the different classifiers on the original training sets (for a baseline) and the oversampled training sets and evaluated the obtained model on the corresponding test sets according to the rules of repeated cross-validation. Among the classifiers, the kNN was performed with *k* = 5 and 7, using both voting schemes mentioned in Section 2.2.1. The SVM was used with linear kernels and with regularization parameters of 1 and 10. The MLP network contained one hidden layer where the number of the folds was *D*, 0.5D, and 0.1D for the different classifier instances, where *D* is the number of the features of the samples. From the family of decision tree classifiers, the Classification and Regression Trees [49] were used with Gini-impurity and entropy classification criteria, with no limit on the height of the trees and also with a limit of 3 and 5. The best result (highest *F*1, *AUC*, *G*, and *Acc*) among the different parameterizations of the classifiers was taken into account for each oversampled data set.

The statistical analysis of the results was performed separately for the data sets belonging to KEEL and the synthetic data sets, and in some of the tests, we also treated the samplers known from the literature and the generated samplers separately.

### 3.1. Analysis 1: Comparison of the Validation Methods

According to Moreno-Torres et al., DOB-SCV is slightly more effective than SCV [20]. The comparative study by Lopez et al. also indicated that higher *AUC* values can be achieved with DOB-SCV than SCV because DOB produces more homogeneous folds. They also showed that the differences become stronger as the level of imbalance increases [19].

Our first experiment aimed to check whether differences between DOB and SCV can be observed even with the use of different samplers, and, since randomness is an essential element of the samplers, we also wanted to check whether the oversamplers alone—using the same validation method—do not cause significant differences in the estimated performance of the classification models. For this purpose, we experimented with each sampler–classifier pair using the same DOB folds twice. The results of the two runs are referred to as DOB and DOB2.

With a Friedman test, which we chose based on the work of Demšar [52], we examined whether the result of the validation methods (the performance score of the models) can be considered the same. Based on the test results, we also rejected the null hypotheses about the same effect for the *F*1, *AUC*, *G*, and *Acc* measures. Next, we applied Nemenyi’s post-hoc test [53], which showed no significant difference in the performance score of the sampler–classifier pairs for DOB and DOB2. However, the results obtained for DOB and SCV folds showed a statistically significant difference (α=0.001). Thus, the findings of Lopez et al. are also valid when different samplers are used and not only for the AUC.

**Figure 1:**
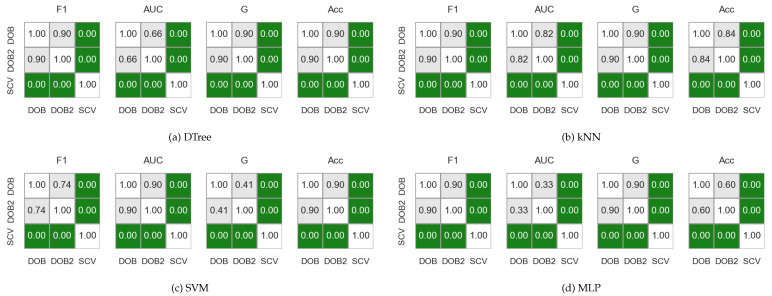
The result of the Nemenyi-test used to compare the classification performance scores obtained with different validations on the KEEL data sets. The oversampling was done by the named samplers.

Figure 1 shows the results obtained on the KEEL data sets with the named samplers, but the statements are valid for both the KEEL and the synthetic data sets regardless of whether we used the named samplers or the generated ones.

### 3.2. Correlation Analysis

**Figure 2:**
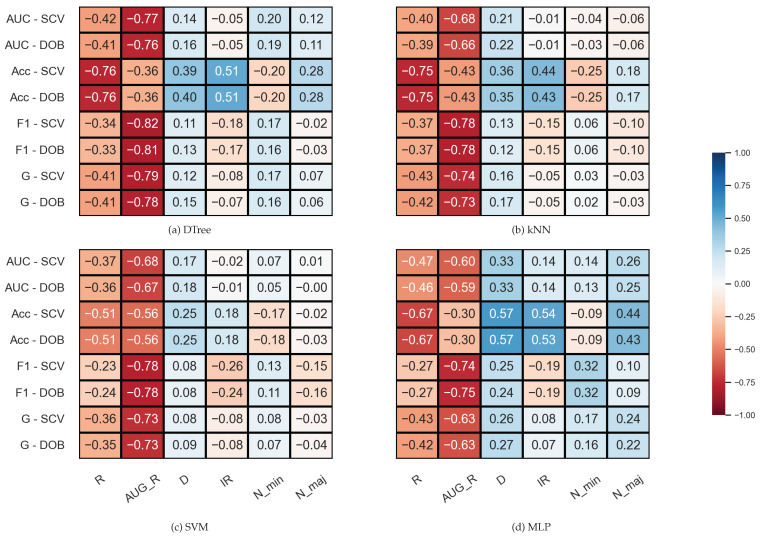
Result of the Spearman’s rank correlation test between some properties of the KEEL data set and the results of the classification combined with oversampling obtained on the data sets.

We examined the correlation between some properties of the data sets and the results (*F*1, *AUC*, *G*, *Acc*) achieved by the sampler–classifier pairs on the data sets. The distributions cannot be considered normal, so we performed Spearman’s rank correlation analysis [54]. For the KEEL data sets, the selected properties were the number of minority (*N_min*) and majority samples (*N_maj*), the imbalance ratio (IR), the number of features (*D*), and two measures to characterize the level of overlap between classes in the data sets (*R* and *AUG_R*). The *R-value* of a data set consisting of two classes is the proportion of elements in the data set that have more than θ elements belonging to the other class among their *k* nearest neighbors. The augmented *R-value* (*AUG_R*) is a version of the *R-value* that considers the size of the classes. The analysis was performed with k=5, θ=2. The results are shown in Figure 2.

One can observe in Figure 2 that there are monotonic relationships of similar strength and direction between the characteristics of the data set and the results of the classification obtained with DOB-SCV and SCV. Most of the database properties show a weak or moderate correlation with the classification performance—the exceptions being the two measures used to describe the overlap of the classes. While *R* shows a strong inverse relationship with *Acc*, the *AUG_R* value designed for imbalanced data sets shows a strong negative correlation with the other scores. Knowing the weaknesses of the *Acc*, it is not surprising that it correlates with the *IR* value more strongly than the other measures. It is more remarkable that the *F*1 also has a moderate negative correlation with the *IR* for all classifiers, although *F*1 is one of the most commonly used measures for imbalanced data sets [55].

**Figure 3:**
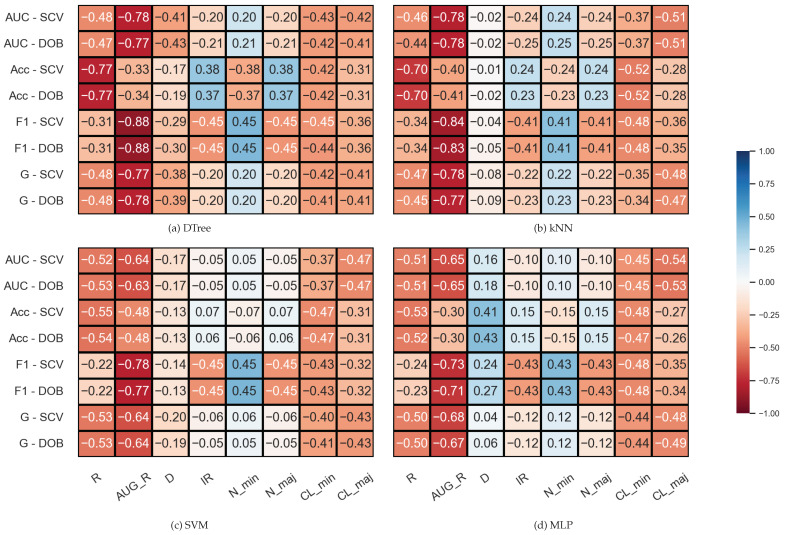
Result of the Spearman’s rank correlation test between some properties of the synthetic data set and the results of the classification combined with oversampling obtained on the data sets.

The results belonging to the synthetic data sets were analyzed similarly, but since the number of clusters within the classes is also known for these data sets, two columns were added to the correlation matrices. The results are shown in Figure 3. We note that the number of samples was fixed during the experiments; the *IR* value determines the *N_min* and *N_maj* values here, which is, of course, also reflected in the correlations. It is also worth noting that the properties of the data set are less diverse than the KEEL’s collection (Table 1).

By comparing the DOB and SCV rows of the correlation matrices, we can see that the values are similar for the two validation methods, and this time again, the *AUG_R* shows a strong negative relationship with the *F*1, *AUC*, and *G* scores regardless of the classification method and there is a negative correlation between the number of clusters appearing within the classes and the results achieved by the classifiers.

We focused on the differences between the validation methods during the subsequent analysis. As mentioned earlier, according to Lopez et al.’s observation, the more imbalanced the data set, the more significant the relative difference between the *AUC* obtained by the DOB-SCV and SCV techniques [19].

To verify that this statement can be considered valid, even with the use of different samplers, we performed a second test to examine the correlation between the properties of the data sets and the relative differences of the validation methods for each measure. The relative difference between the two validation techniques was determined for each data set as specified based on the formula provided by the authors in Ref. [19],

(5) $${\mathrm{diff}}_{V}=\frac{V_{DOB\_SCV}-V_{SCV}}{V_{scv}}\;,$$

where $V_{DOB\_SCV}$ and $V_{SCV}$ are the mean performance scores of a sampler–classifiers estimated by the DOB-SCV and SCV methods, respectively (V∈{F1,AUC,G,Acc}).

**Figure 4:**
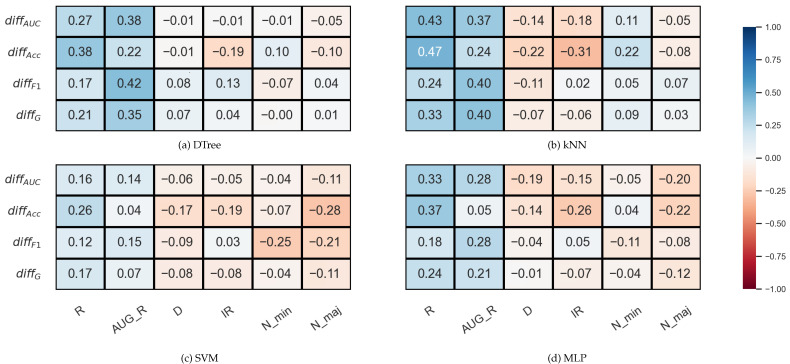
Result of the Spearman’s rank correlation test between some features of the KEEL data set and the difference of the DOB and SCV results of the classification combined with oversampling obtained on the data sets.

In Figure 4, which shows the results for the KEEL data set, we can immediately notice that there are no strong correlations between the data set properties and the relative difference of the validation methods.

In the case of the decision tree and the SVM, there is practically no correlation between the IR and the ${\mathrm{diff}}_{\mathrm{AUC}}$. In the case of kNN and MLP, a weak negative correlation can be observed, as well as between the *IR* and the relative difference of the *Acc* values. However, the relative differences show a moderately strong positive correlation with the *R* or *AUG_R* values in the case of DTree, kNN, and MLP.

**Figure 5:**
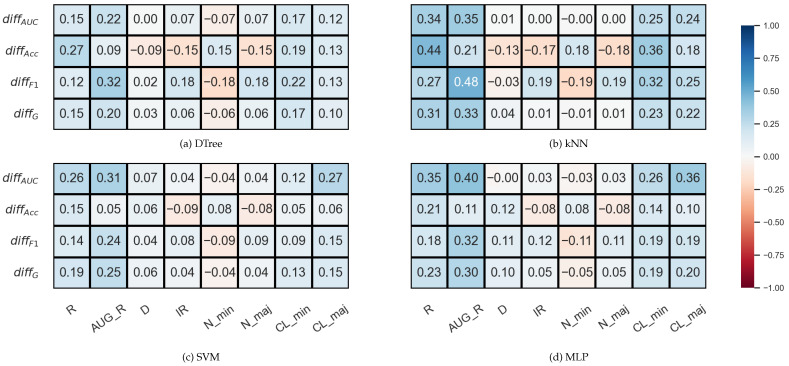
Result of the Spearman’s rank correlation test between some features of the synthetic data set and the relative differences between the DOB-SCV and SCV results of the classification combined with oversampling obtained on the data sets.

In the case of the synthetic data sets, the correlations between *IR* and the ${\mathrm{diff}}_{\mathrm{AUC}}$ can be considered neutral rather than negative (Figure 5). However, note that the IR values in the synthetic data set are not nearly as diverse as in the case of KEEL.

Additionally, we can see again that the relative differences show a positive correlation with the degree of overlap between the classes to a greater or lesser degree, and also with the number of clusters within the classes.

Although the observation of Lopez’s et al. on the relationship between IR and ${\mathrm{diff}}_{\mathrm{AUC}}$ could not be confirmed in our experiments, we have found other database properties worth paying attention to when choosing between DOB and DOB-SCV validation.

### 3.3. Graphical Analysis per Data Set

To see behind the numbers, we visualized the relationship between the validation methods and the performance scores of the sampler–classifier pairs on violin plots. The shape of the “violin body” is determined by the distribution of the results obtained for a particular data set by a certain classifier combined with different oversamplers. The measure was also fixed. The red and the black lines in the diagrams show the mean performance of the classifier without sampling (ws) and with sampling, respectively.

**Figure A1:**
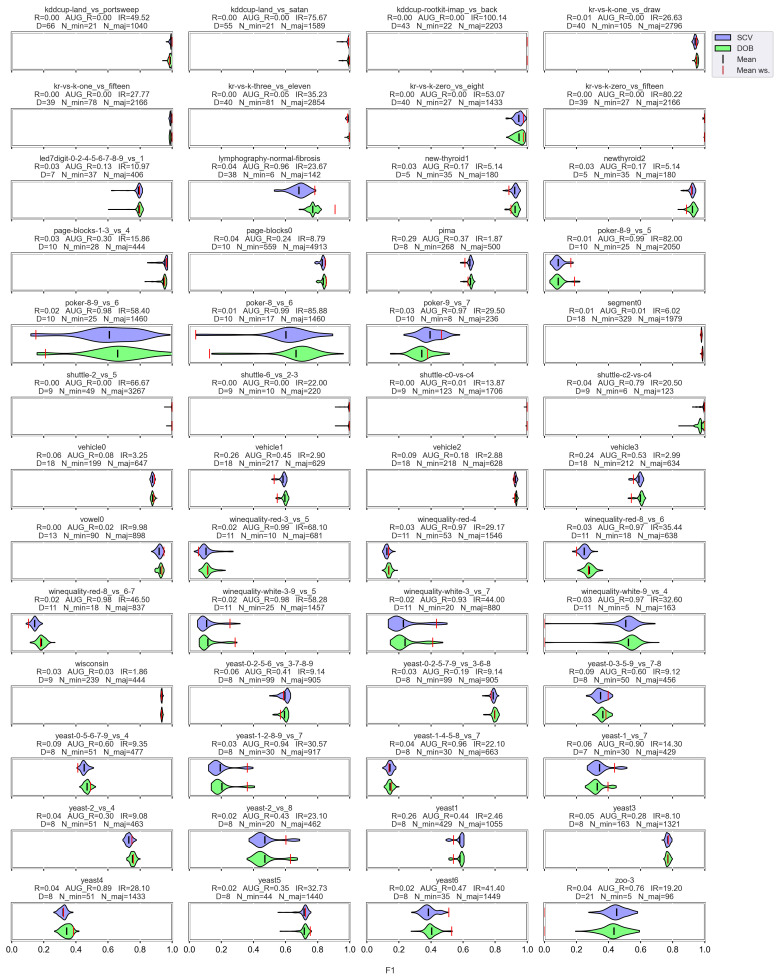
*F*1 values resulted by DTree classifier and named oversamplers per data set of KEEL.

**Figure A2:**
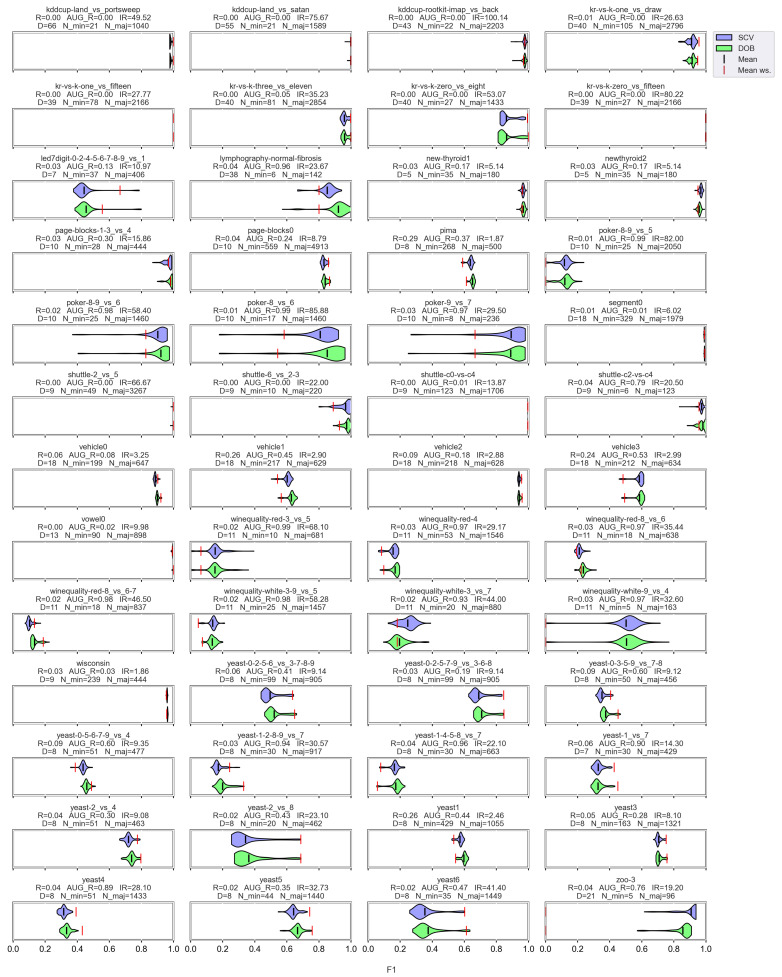
*F*1 values resulted by the kNN classifier and the named oversamplers per data set of KEEL.

**Figure A3:**
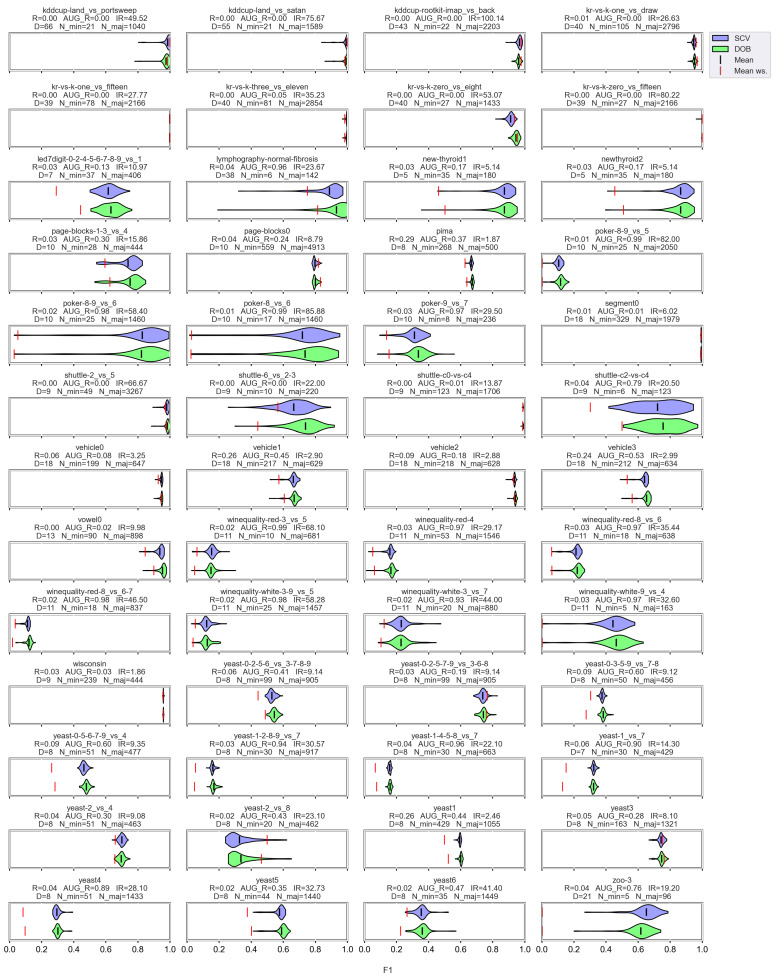
*F*1 values resulted by the MLP classifier and the named oversamplers per data set of KEEL.

**Figure A4:**
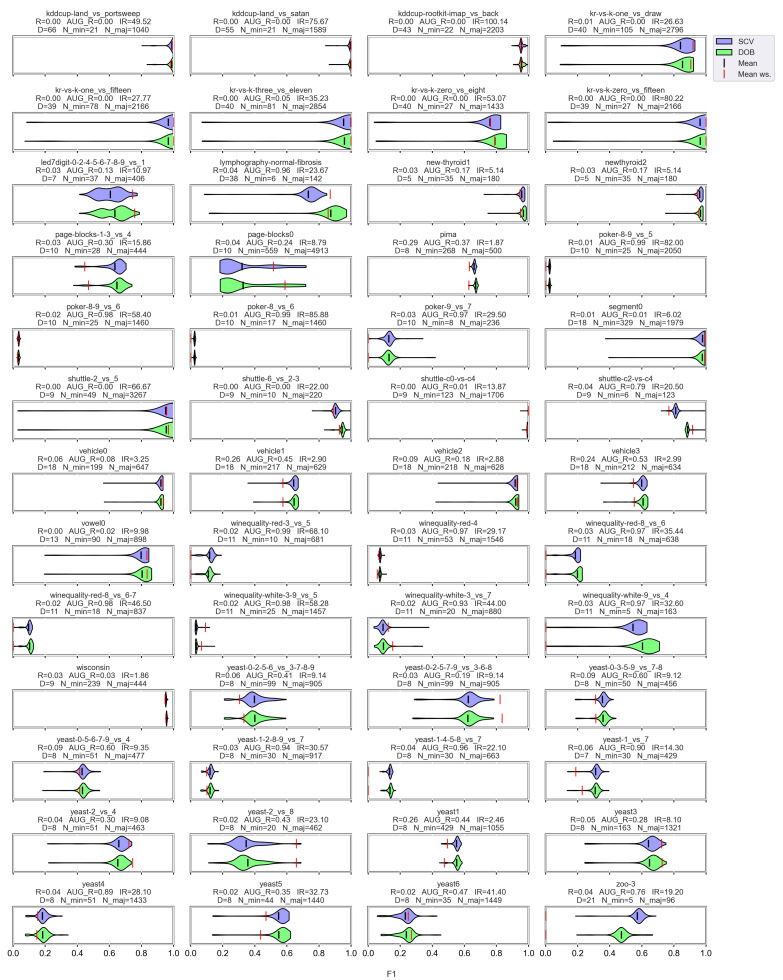
*F*1 values resulted by the SVM classifier and the named oversamplers per data set of KEEL.

Based on the literature, the nature of the data determines which classifier can be used more successfully without applying any oversampling [56,57]. It also affects the selection of the appropriate oversampler [40]. The violin plots also show the importance of choosing the right classifier and sampler. (Figure A1, Figure A2, Figure A3 and Figure A4). We can observe several data sets where applying the appropriate oversampler before classification significantly improves, while a poorly selected sampler worsens the results. On the other hand, some data sets can be classified well without oversampling. For example, the *winequality-white-3-9_vs_5* (Figure A1).

The difference in the effect of validation is less striking; however, there is a slight but relatively stable difference between them (Appendix A). Stable, in the sense that not only the mean performance scores of the classifiers are similar, but also the distribution of the results achieved with different oversamplers in most cases. Regardless of whether we divided the data set according to the DOB or SCV partitioning, the classification improved with a similar number of oversamplers.

**Figure 6:**
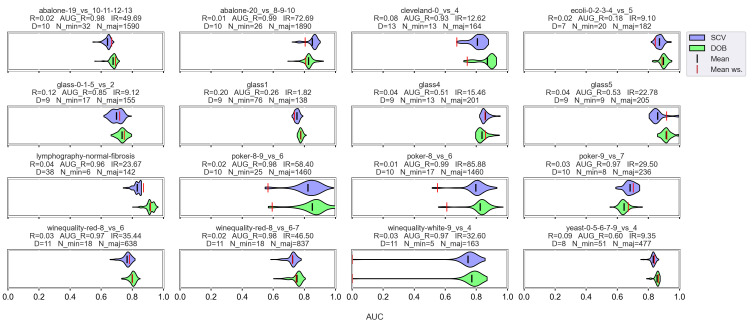
*AUC* values achieved by the named samplers and DTree classifiers on KEEL data sets where the largest relative differences were measured.

However, let us see some examples where differences arose. Figure 6 shows a few selected KEEL data sets where the mean of the *AUC* values achieved by variously named samplers and DTree classifier differ the most using the two validation methods.

Note that the *R_AUG* values are quite high for most of these data sets, and there are a few cases where the number of minority samples is extremely small. When these two things happen simultaneously, it is difficult to predict the outcome. For example, on the lymphography-normal-fibrosis data set, the SCV partitioning provides such folds that oversampling could only worsen the initial result. On the other hand, we can see the *poker-9_vs_7* data sets where the classification performance improved in more cases when we sampled the folds generated by SCV than by DOB. In our opinion, the location of the samples plays a crucial role, which is not described by many measures. Furthermore, selecting the most representative samples from the clusters has a significant role in overlapping classes.

For each classifier, we can find examples where there are larger differences between the two types of validation, but these differences of a few percent are, in many cases, insignificant compared to the effect of the oversampling.

Table A1 and Table A2 show how much the validation meant for each sampler on average for the KEEL and for the synthetic data set for each sampler.

### 3.4. Graphical Analysis for Clusters

Earlier, we saw that the number of clusters within the classes shows a negative correlation with the classification result and a positive correlation with the relative differences of the validation methods (${\mathrm{diff}}_{V}$). This section aims to investigate the visualized connections between the number of clusters in the majority or minority classes and several metrics.

**Figure 7:**
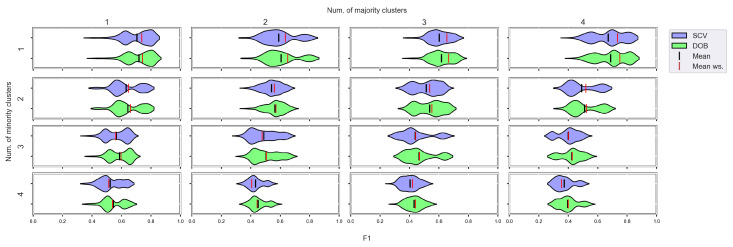
*F*1 values concerning the number of clusters in the minority and majority classes in the case of the DTree classifier. (IR8).

**Figure 8:**
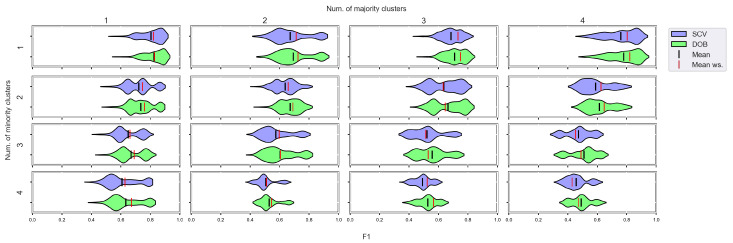
*F*1 values concerning the number of clusters in the minority and majority classes in the case of the kNN classifier. (IR8).

**Figure 9:**
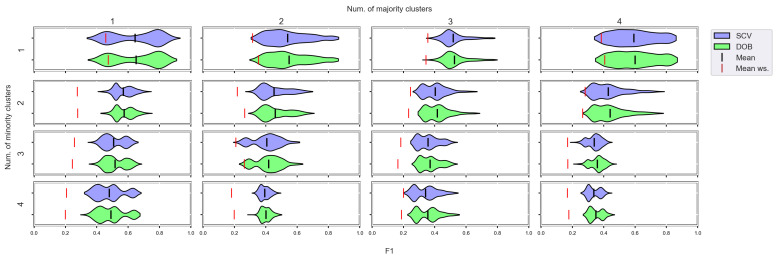
*F*1 values concerning the number of clusters in the minority and majority classes in the case of the MLP classifier. (IR8).

**Figure 10:**
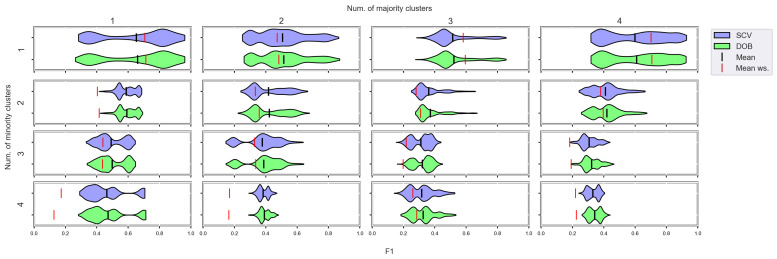
*F*1 values concerning the number of clusters in the minority and majority classes in the case of the SVM classifier. (IR8).

We again used violin-plots to represent the results of our experiments, but the data has now been plotted in a grouped form. The values on synthetic data sets with the same number of minority and majority clusters were placed on the sample plot. The plots belong to different classifiers, and different metrics were placed in different figures (Figure 7, Figure 8, Figure 9 and Figure 10).

We can see the results obtained with the generated samplers from the synthetic data sets. It can be noticed that even despite the large number of oversampling methods, the distribution of the performance scores estimated by DOB and SCV is very similar.

Compared to the classification without oversampling, the classification combined with oversampling is significantly better when the number of clusters of the minority class increases. On the other hand, the change in the number of clusters of the majority class has less influence on the efficiency in terms of *F*1 of the classifications combined with oversampling.

It is interesting to note that the performance of the SVM and the MLP concerning *F*1 decreased radically, regardless of the validation method when the number of the minority clusters was increased and smaller clusters were formed. The large *IR* alone does not cause problems in classification, but difficulties can arise if classes overlap. It is worth noting that we found a stronger relationship between the *R* values and the *F*1 values—for both validations—than between the number of clusters and the *F*1 value.

**Figure A9:**
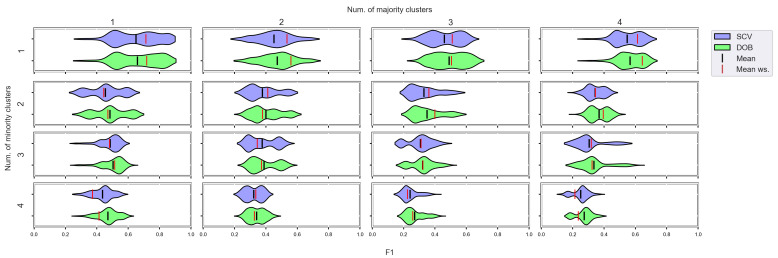
*F*1 values concerning the number of clusters in the minority and majority classes in the case of the DTree classifier. (IR16).

**Figure A10:**
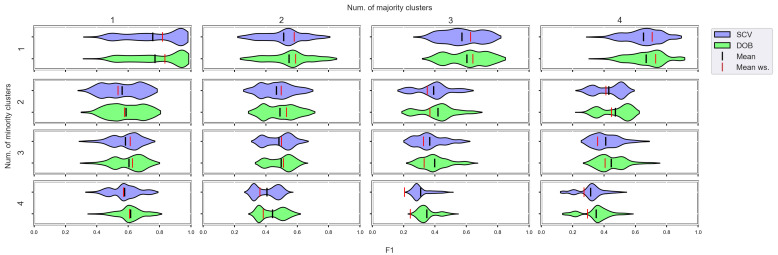
*F*1 values concerning the number of clusters in the minority and majority classes in the case of the kNN classifier. (IR16).

**Figure A11:**
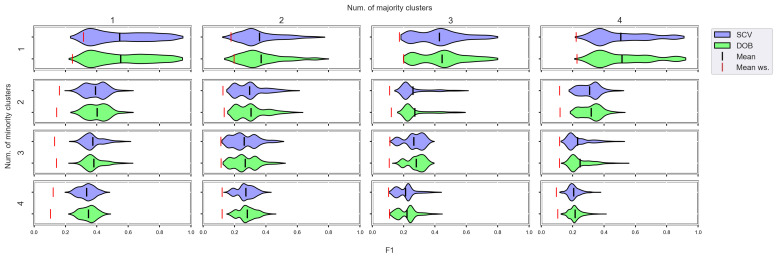
*F*1 values concerning the number of clusters in the minority and majority classes in the case of the MLP classifier. (IR16).

**Figure A12:**
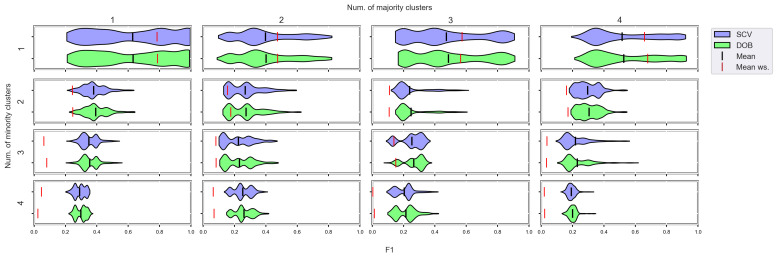
*F*1 values concerning the number of clusters in the minority and majority classes in the case of the SVM classifier. (IR16).

Based on our experiments, we cannot confirm the statement [19] that the difference between the two verification techniques involved in our tests (SCV, DOB-SCV) increases as the imbalance ratio of the data sets increases. Figure A9, Figure A10, Figure A11 and Figure A12 are the same figures with a higher imbalance ratio (IR16 instead of IR8). Our measurements only show that the SVM and the MLP without oversampling perform better when the imbalance ratio is low, which is most likely explained by the fact that more elements can be found in each cluster of the minority class than when the imbalance rate is high.

The classification combined with oversampling is significantly better when the number of clusters of the minor class increases. See Figure A9, Figure A10, Figure A11 and Figure A12.

## 4. Conclusions

We have published the result of our extensive study involving 420 data sets about the SCV and the DOB-SCV methods when oversampling is used before classification. We have verified the differences between DOB-SCV and SCV with a suitable statistical test, examined the correlation between some properties of the data sets and the values *F*1, *AUC*, *G*, *Acc* achieved by the sampler–classifier pairs on the data sets, and conducted visual data analysis.

We would like to summarize the most important results below:

- In general, slightly higher *F*1, *AUC G*, *Acc* values can be achieved with DOB-SCV than SCV in combination oversamplers and classifiers;
- Based on our experiments, we can not confirm the statement [19] that the difference between the two verification techniques involved in our tests (SCV, DOB-SCV) increases when the imbalance ratio of the data sets increases;
- We can state that there is a difference between SCV and DOB-SCV in favor of DOB-SCV when the number of clusters within the classes or the volume of overlapping between the clusters increases;
- The selection of the sampler–classifier pair is much more critical for the classification performance than the choice between these two validation techniques.

Our results could help researchers to focus on the part of the training process that can significantly impact classification performance and to choose the right validation method for the given situation. 

## Figures and Tables

**Table 1:** The range of properties of the database collections.

	R	AUG_R	D	IR	N_min	N_maj
KEEL	0.0000–0.2851	0.0000–0.9923	3–66	1.8157–129.4375	5–559	83–4913
Synthetic	0.0083–0.1083	0.1305–0.9403	4; 8	7.9552; 15.6667	36; 67	533; 564

**Figure A5:**
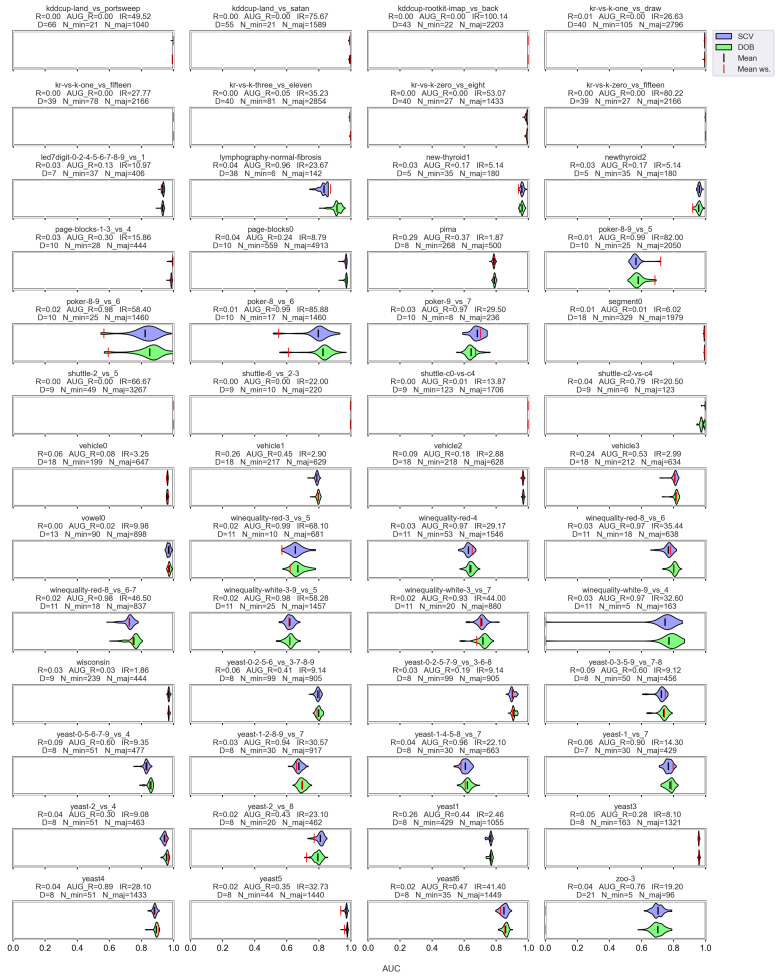
*AUC* values resulted by the DTree classifier and the named oversamplers per data set of KEEL.

**Figure A6:**
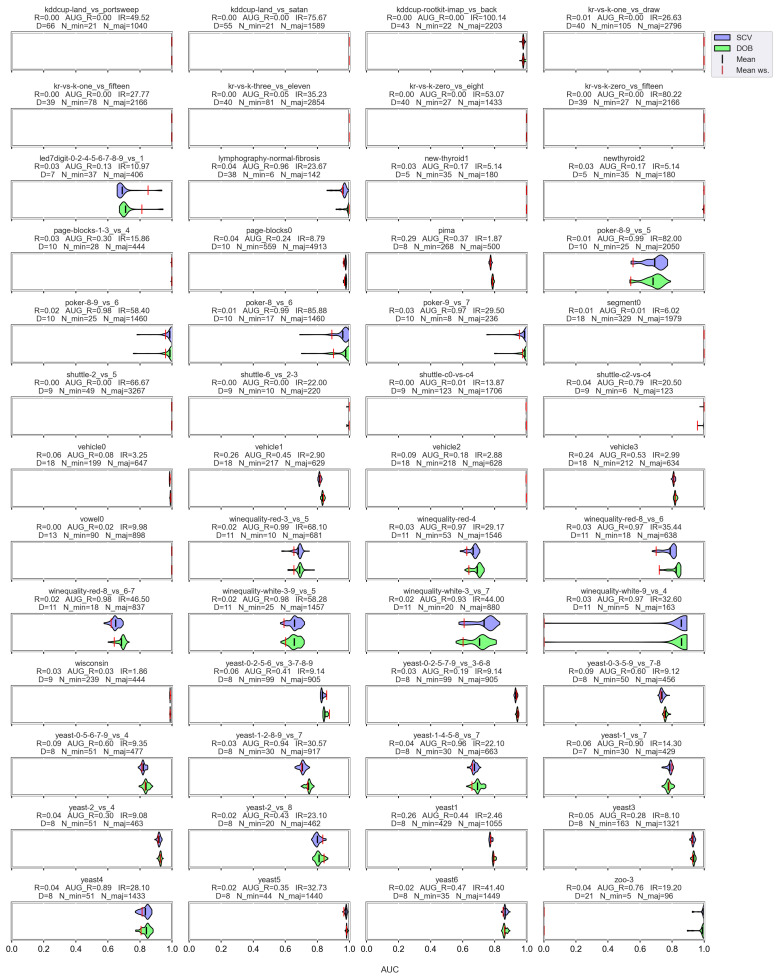
*AUC* values resulted by the kNN classifier and the named oversamplers per data set of KEEL.

**Figure A7:**
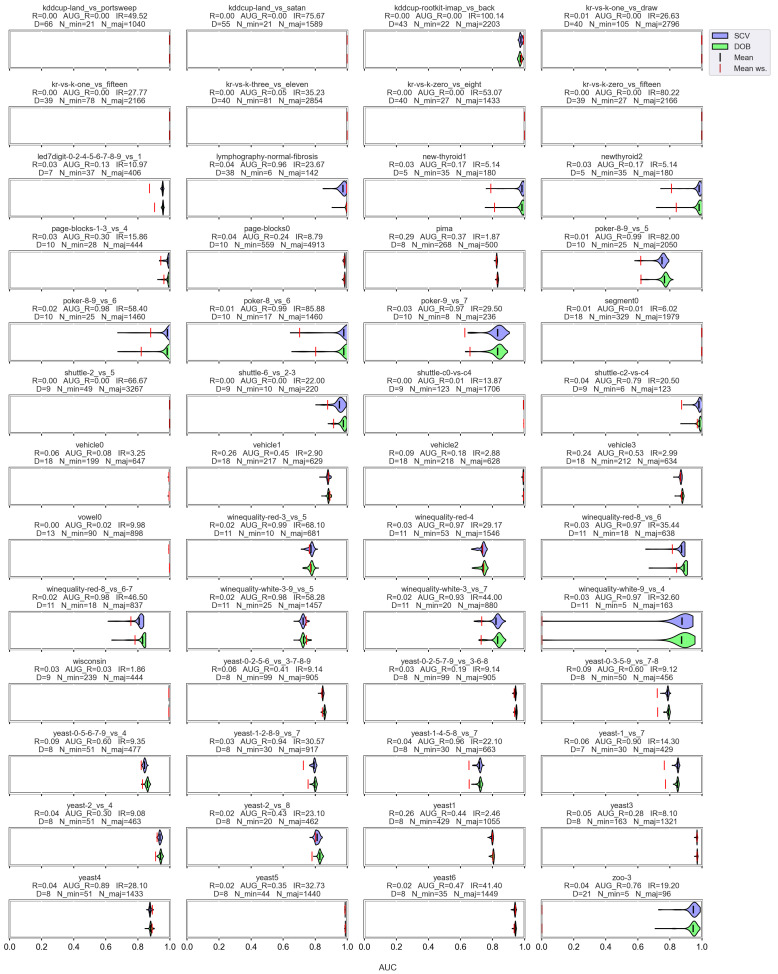
*AUC* values resulted by the MLP classifier and the named oversamplers per data set of KEEL.

**Figure A8:**
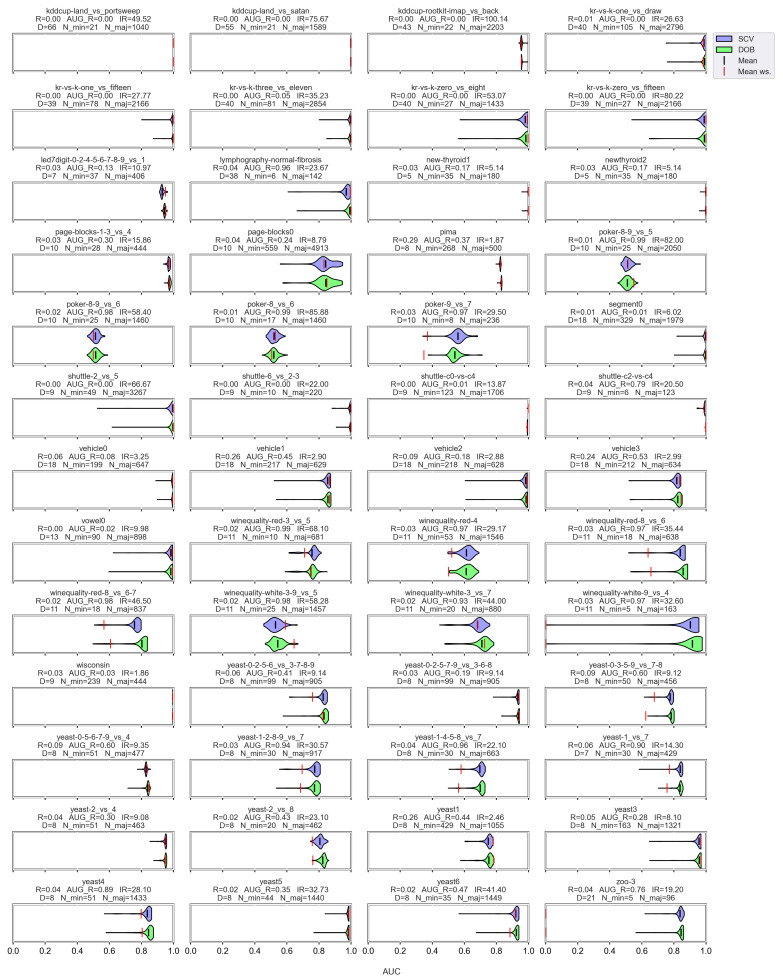
*AUC* values resulted by the SVM classifier and the named oversamplers per data set of KEEL.

## Data Availability

The data sets used in this study are available at the UCI Machine Learning Repository at https://archive.ics.uci.edu/ml/datasets.php (accessed on 10 December 2022) or KEELdataset repository at https://keel.es (accessed on 10 December 2022). The synthetic data generated by the authors are available at https://github.com/szghlm/SyntImbData (accessed on 10 December 2022).

## Appendix A

The results of the experiments performed on the data sets of the KEEL in relation to *F*1 presented per classifier.

## Appendix B

The results of the experiments performed on the data sets of the KEEL in relation to *AUC* presented per classifier.

## Appendix C

*F*1 values concerning the number of clusters in the minority and majority classes per classifier. The imbalance rate of the used data sets is 16.

## Appendix D

This section contains tables showing the relative difference of the validation methods in aggregate per sampler. The results for the 460 generated samplers are summarized under the name ModularOverSampler.

**Table A1:** Mean of the relative differences of the DOB-SCV and SCV per oversampler on the KEEL data sets.

	DTree				kNN			
Sampler	**%** ${\mathrm{diff}}_{\mathrm{AUC}}$	**%** ${\mathrm{diff}}_{\mathrm{Acc}}$	**%** ${\mathrm{diff}}_{F1}$	**%** ${\mathrm{diff}}_{G}$	**%** ${\mathrm{diff}}_{\mathrm{AUC}}$	**%** ${\mathrm{diff}}_{\mathrm{Acc}}$	**%** ${\mathrm{diff}}_{F1}$	**%** ${\mathrm{diff}}_{G}$
Assembled-SMOTE	0.79	0.21	1.16	0.65	0.89	0.36	1.65	0.97
CCR	0.70	0.14	1.36	0.96	1.02	0.29	1.93	1.21
G-SMOTE	1.05	0.26	1.91	1.10	0.89	0.34	1.83	1.09
LVQ-SMOTE	0.57	0.27	1.49	0.58	0.89	0.39	2.27	1.32
Lee	0.98	0.26	1.71	0.87	0.87	0.38	1.61	0.97
ModularOverSampler	0.71	0.26	1.49	0.92	0.96	0.39	2.06	1.37
ProWSyn	0.93	0.23	1.27	0.95	0.86	0.43	1.73	1.00
Polynomial fitting SMOTE	0.80	0.14	1.47	1.11	0.95	0.31	2.09	1.31
SMOBD	0.86	0.23	1.44	0.82	0.85	0.35	1.47	0.89
SMOTE	0.96	0.28	1.69	0.79	0.86	0.34	1.57	0.96
SMOTE-IPF	0.92	0.24	1.21	0.86	0.85	0.37	1.54	0.90
SMOTE-TomekLinks	0.91	0.19	1.21	0.94	0.90	0.35	1.61	0.99
	SVM				MLP			
Assembled-SMOTE	0.48	0.29	1.70	0.54	0.52	0.18	1.16	0.53
CCR	0.64	0.26	1.19	0.56	0.65	0.18	1.00	0.64
G-SMOTE	0.48	0.24	1.73	0.73	0.51	0.18	1.15	0.68
LVQ-SMOTE	0.46	0.12	1.04	0.48	0.50	0.21	1.00	0.56
Lee	0.46	0.18	1.61	0.65	0.51	0.19	1.19	0.55
ModularOverSampler	0.41	0.25	1.70	0.63	0.59	0.27	1.42	0.69
ProWSyn	0.45	0.24	1.49	0.54	0.55	0.25	1.36	0.68
Polynomial fitting SMOTE	0.45	0.14	1.44	0.50	0.58	0.27	1.46	0.64
SMOBD	0.43	0.11	1.49	0.48	0.49	0.23	1.34	0.68
SMOTE	0.38	0.28	1.54	0.49	0.83	0.37	1.54	0.78
SMOTE-IPF	0.44	0.32	1.50	0.42	0.61	0.29	1.24	0.57
SMOTE-TomekLinks	0.45	−0.06	1.69	0.53	0.59	0.23	1.23	0.79

**Table A2:** Mean of the relative differences of the DOB-SCV and SCV per oversampler on the synthetic data sets.

	DTree				kNN			
Sampler	**%** ${\mathrm{diff}}_{\mathrm{AUC}}$	**%** ${\mathrm{diff}}_{\mathrm{Acc}}$	**%** ${\mathrm{diff}}_{F1}$	**%** ${\mathrm{diff}}_{G}$	**%** ${\mathrm{diff}}_{\mathrm{AUC}}$	**%** ${\mathrm{diff}}_{\mathrm{Acc}}$	**%** ${\mathrm{diff}}_{F1}$	**%** ${\mathrm{diff}}_{G}$
Assembled-SMOTE	1.77	0.61	4.49	1.73	1.43	0.81	4.62	1.85
CCR	1.58	0.55	4.09	1.52	1.58	0.78	4.93	2.01
G-SMOTE	1.86	0.54	4.68	1.86	1.53	0.70	4.61	1.98
LVQ-SMOTE	1.48	0.64	4.02	1.49	1.46	0.74	4.35	1.77
Lee	1.80	0.61	4.49	1.77	1.44	0.79	4.53	1.86
ModularOverSampler	1.80	0.59	4.59	1.97	1.65	0.82	4.97	2.24
ProWSyn	1.80	0.71	4.19	1.77	1.45	0.98	4.57	1.79
Polynomial Fitting SMOTE	1.49	0.41	4.33	1.97	1.54	0.56	4.70	2.33
SMOBD	1.79	0.61	4.46	1.74	1.44	0.80	4.52	1.83
SMOTE	1.86	0.63	4.68	1.77	1.42	0.77	4.49	1.86
SMOTE-IPF	1.79	0.65	4.74	1.74	1.46	0.80	4.55	1.87
SMOTE-TomekLinks	1.77	0.62	4.65	1.83	1.48	0.81	4.60	1.86
	SVM				MLP			
Assembled-SMOTE	1.55	0.47	2.44	1.42	1.45	0.56	3.13	1.62
CCR	1.43	0.41	2.11	1.16	1.25	0.49	2.58	1.36
G-SMOTE	1.50	0.43	2.41	1.35	1.40	0.41	3.05	1.52
LVQ-SMOTE	1.32	0.47	2.10	1.14	1.26	0.45	2.69	1.28
Lee	1.55	0.47	2.46	1.46	1.43	0.44	2.95	1.60
ModularOverSampler	1.61	0.44	2.53	1.47	1.56	0.49	3.34	1.70
ProWSyn	1.48	0.41	2.27	1.29	1.36	0.52	2.88	1.46
Polynomial Fitting SMOTE	1.42	0.24	2.38	1.35	1.43	0.39	2.95	1.36
SMOBD	1.51	0.40	2.32	1.36	1.41	0.48	2.97	1.58
SMOTE	1.56	0.50	2.41	1.45	1.48	0.53	2.93	1.55
SMOTE-IPF	1.58	0.45	2.39	1.43	1.40	0.47	3.06	1.67
SMOTE-TomekLinks	1.54	0.34	2.31	1.38	1.55	0.69	3.11	1.57
